# Fecal Bacteriotherapy: A Case Report in an Immunosuppressed Patient with Ulcerative Colitis and Recurrent  *Clostridium difficile* Infection

**DOI:** 10.1155/2012/810943

**Published:** 2012-04-23

**Authors:** Hadeel Zainah, Ann Silverman

**Affiliations:** ^1^Infectious Diseases Division, Henry Ford Hospital, 2799 West Grand Boulevard, CFP-304, Detroit, MI 48202, USA; ^2^Division of Gastroenterology, UP Digestive Health, War Memorial Hospital, Medical Office Building (NEW), 509 Osborn Boulevard, Suite 340, Sault Sainte Marie, MI 49783, USA

## Abstract

We report a case of ulcerative colitis (UC) and recurrent *Clostridium difficile* infection (CDI) where the patient was on immunomodulatory therapy and had successful CDI eradication after fecal transplantation. This is the first case report in the literature documenting successful *C. difficile* eradication in an immunosuppressed patient. We feel that fecal transplantation should be studied as a treatment option in these patients.

## 1. Introduction

*Clostridium difficile *has emerged as a major cause of antibiotic-associated diarrhea [1]. The treatment of recurrent *Clostridium difficile *infection (CDI) has not been standardized; however, fecal bacteriotherapy has demonstrated an average success rate of 92% as shown in a recent systemic review [2].

Association between CDI and ulcerative colitis (UC), which is an inflammatory bowel disease with unknown etiology, has been reported [3, 4]; however, fecal bacteriotherapy has been performed particularly in few patients with inflammatory bowel disease and none while the patient is on immunosuppression.

## 2. Case Presentation

**Figure 1:**
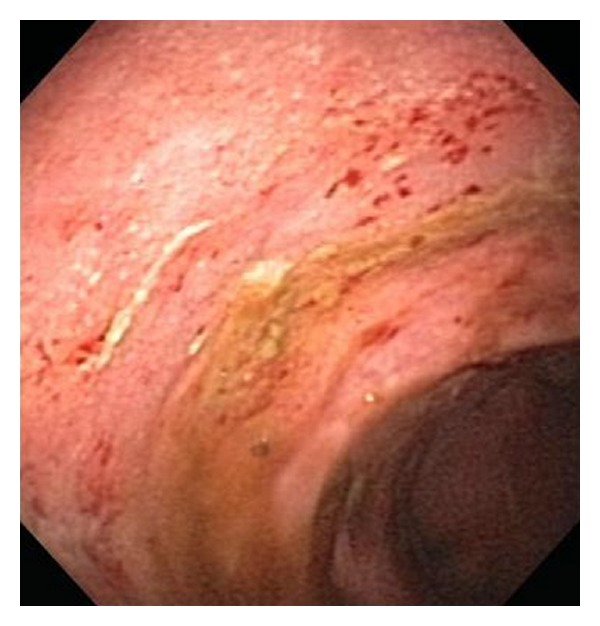
Picture of the patient's colon taken during colonoscopy. The image reveals diffuse inflammation with submucosal hemorrhage.

The patient is a 51-year-old man with a 13-year history of UC, who experienced frequent exacerbations of his disease proven by colonoscopies (Figure 1) and subsequent biopsies. Some of the flares were severe and some of them were mild to moderate. Early in the course of the disease, the patient was treated with three doses of infliximab; however, this treatment was terminated based on the patient's decision. He did not tolerate aminosalicylic acid derivatives due to an allergic reaction; therefore, azathioprine was used chronically to suppress the UC in addition to episodic oral or rectal steroids to treat the breakthrough flares. The azathioprine dose ranged mainly between 100–125 mg daily.

Most exacerbations were related to CDI, which was diagnosed by testing the stool for *Clostridium difficile* toxin (PCR was used once to establish the diagnosis). CDI recurred 4 times in the last few years, which made it more difficult to control the UC. The patient was complaining of continuous weakness and fatigue along with the diarrhea. CDI episodes were treated with oral vancomycin. The eradication of CDI allowed better control of his UC; therefore, he was committed to long-term suppressive therapy with oral once-daily vancomycin.

Fecal transplantation was considered in this patient despite the uniqueness of this case. The risks and benefits were discussed with the patient. His blood work prior to the procedure revealed: white blood count of 5.7 k/uL, hemoglobin of 15.5 mg/dL, platelets of 258 k/uL, Albumin of 4.2 mg/dL, and creatinine of 1.1 mg/dL. The donor's stool specimen was obtained from the wife who tested negative for *Clostridium difficile *PCR in the stool. Also, the wife serology was negative for HIV and hepatitis (hepatitis A antibodies, hepatitis B surface antigen, hepatitis B core antibodies, and hepatitis C antibodies). Oral vancomycin was discontinued 24 hours before the transplantation. The stool suspension was prepared according to standard protocols [5]. Warm water was added to 300 mL of the donor's stool, and the sample was instilled via colonoscope without complications. Afterwards, the patient remained symptom-free for 8 months and was able to stop oral vancomycin without CDI recurrence to date. The procedure was not associated with side effects despite the fact that it was done while the patient was on an immunomodulator. The patient was aware of the unusual nature of his circumstance and was anxious to have this information available for other patients with similar problem.

## 3. Discussion

Treatment of recurrent CDI with vancomycin and metronidazole has not always been successful. Case reports of fecal bacteriotherapy have reported a high rate of *C. difficile* eradication based on the fact that restoration of the normal colonic flora helps to eradicate *Clostridium difficile *[6]. The experience with this evolving therapy is limited but a recent case-series of 100 patients (without UC) showed 90% cure rate associated with fecal transplantation [7].

Clinical outcomes can be worse when CDI coincides with UC flares compared to non-CDI-associated flares increasing the rate of surgery [8], length of hospital stay and, mortality [9].

 Fecal bacteriotherapy has been recently used for the treatment of relapsing UC in selected patients. That was demonstrated in a case-series of 6 patients with confirmed UC who suffered severe recurrent bouts of UC but experienced improvement in some symptoms by 1 week after fecal transplantation and complete remission of UC by 4 months after therapy [10]. Nevertheless, this therapy has not been used for recurrent CDI in UC patients on immunomodulatory therapy.

Our patient had recurrent UC flares associated with CDI, which mandated using oral vancomycin to prevent recurrence. The patient was interested in a long-term cure of CDI through fecal bacteriotherapy. It was a challenge to perform this procedure in this patient since he was on azathioprine but that did not affect the outcome. Both CDI and UC subsided and the patient's fatigue resolved.

This is the first report of bacteriotherapy using a fecal enema in a patient with UC and chronic CDI on immunomodulatory therapy. The observation made from our case suggests that fecal bacteriotherapy might be safe in CDI patients with UC on immunosuppression. Furthermore, the safety profile needs to be evaluated further.
